# A prospective evaluation of thiamine and magnesium status in relation to clinicopathological characteristics and 1-year mortality in patients with alcohol withdrawal syndrome

**DOI:** 10.1186/s12967-019-02141-w

**Published:** 2019-11-21

**Authors:** Donogh Maguire, Dinesh Talwar, Alana Burns, Anthony Catchpole, Fiona Stefanowicz, Gordon Robson, David P. Ross, David Young, Alastair Ireland, Ewan Forrest, Peter Galloway, Michael Adamson, Eoghan Colgan, Hannah Bell, Lesley Orr, Joanna-Lee Kerr, Xen Roussis, Donald C. McMillan

**Affiliations:** 1 Emergency Medicine Department, Glasgow Royal Infirmary, 84 Castle Street, Glasgow, G4 0SF Scotland, UK; 2 Academic Unit of Surgery, School of Medicine, University of Glasgow, New Lister Building, Royal Infirmary, Glasgow, G31 2ER Scotland, UK; 3 The Scottish Trace Element and Micronutrient Diagnostic and Reference Laboratory, Department of Biochemistry, Royal Infirmary, Glasgow, G31 2ER Scotland, UK; 4 Department of Biochemistry, Queen Elizabeth University Hospital, Glasgow, G51 4TF Scotland, UK; 5 Department of Mathematics and Statistics, University of Strathclyde, 26 Richmond Street, Glasgow, G1 1XH Scotland, UK; 6 Department of Gastroenterology, Glasgow Royal Infirmary, Glasgow, G4 0SF Scotland, UK

**Keywords:** Alcohol withdrawal syndrome (AWS), Serum magnesium concentration, Circulating thiamine diphosphate (TDP), Pseudo-hypoxia, Plasma lactate concentrations, Glasgow modified alcohol withdrawal scale (GMAWS), Seizure kindling, 1-year mortality

## Abstract

**Background:**

Alcohol withdrawal syndrome (AWS) is routinely treated with B-vitamins. However, the relationship between thiamine status and outcome is rarely examined. The aim of the present study was to examine the relationship between thiamine and magnesium status in patients with AWS.

**Methods:**

Patients (n = 127) presenting to the Emergency Department with AWS were recruited to a prospective observational study. Blood samples were drawn to measure whole blood thiamine diphosphate (TDP) and serum magnesium concentrations. Routine biochemistry and haematology assays were also conducted. The Glasgow Modified Alcohol Withdrawal Score (GMAWS) measured severity of AWS. Seizure history and current medications were also recorded.

**Results:**

The majority of patients (99%) had whole blood TDP concentration within/above the reference interval (275–675 ng/gHb) and had been prescribed thiamine (70%). In contrast, the majority of patients (60%) had low serum magnesium concentrations (< 0.75 mmol/L) and had not been prescribed magnesium (93%). The majority of patients (66%) had plasma lactate concentrations above 2.0 mmol/L. At 1 year, 13 patients with AWS had died giving a mortality rate of 11%. Male gender (p < 0.05), BMI < 20 kg/m^2^ (p < 0.01), GMAWS max ≥ 4 (p < 0.05), elevated plasma lactate (p < 0.01), low albumin (p < 0.05) and elevated serum CRP (p < 0.05) were associated with greater 1-year mortality. Also, low serum magnesium at time of recruitment to study and low serum magnesium at next admission were associated with higher 1-year mortality rates, (84% and 100% respectively; both p < 0.05).

**Conclusion:**

The prevalence of low circulating thiamine concentrations were rare and it was regularly prescribed in patients with AWS. In contrast, low serum magnesium concentrations were common and not prescribed. Low serum magnesium was associated more severe AWS and increased 1-year mortality.

## Background

Despite public health measures, alcohol consumption has continued to rise in the USA and UK over the past three decades [1–3]. In the USA, the number of alcohol related deaths is reported to have increased by 35% between 2007 and 2017 [4], while Scotland is reported to have the highest alcohol related death rate in the UK [5]. Despite these recent epidemiological reports, there is a paucity of data available in the literature related to mortality for patients following an episode of alcohol withdrawal syndrome (AWS) [6].

Alcohol exerts direct and indirect effects on cellular energy metabolism and alcohol use disorder (AUD) is a complex psychological and pathophysiological problem [7, 8]. Nutritional and social factors may be protective or contributory, however a threshold may be reached beyond which alcohol related compromise of oxidative resilience manifests in accelerated organ/system specific final common pathways of biochemical and clinical deterioration [7, 9]. The occurrence of alcohol withdrawal syndrome (AWS) may represent the crossing of that threshold [9].

**Fig. 1:**
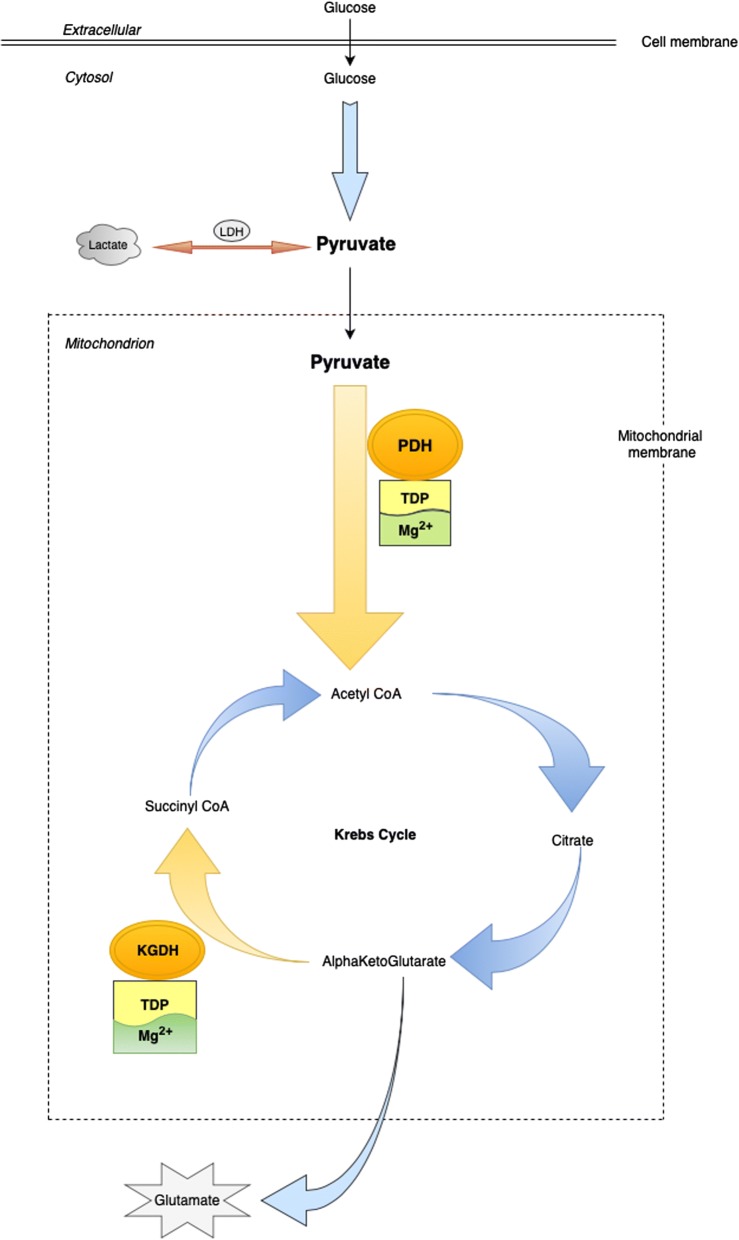
‘Clean burn’ metabolism with normal lactate and glutamate production

**Fig. 2:**
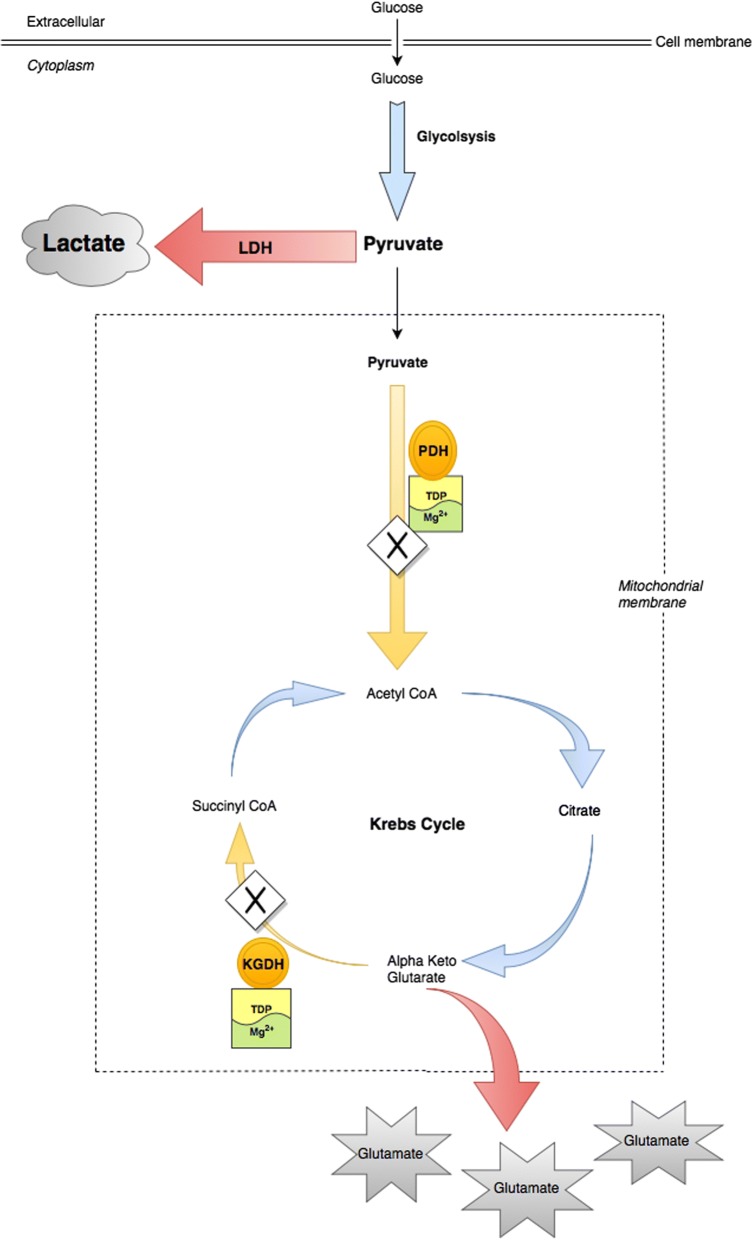
‘Dirty burn’ metabolism: Pseudo-hypoxic dysregulation of glucose metabolism with increased production of lactate and excitatory glutamate in patients with AWS

Standard treatment of AWS is predicated upon early provision of benzodiazepine treatment to ameliorate symptoms and prevent progression to AWS seizures, mainly by restoring the balance between the N-methyl-d-aspartate (NMDA) and gamma-aminobutyric acid (GABA) receptor activity at the synapse [10, 11]. Benzodiazepines replicate the GABA enhancing effect of alcohol on the CNS, thus down-regulating excitatory NMDA activity and preventing progression of alcohol withdrawal syndrome and seizures [10, 11]. In addition, there is empirical provision of thiamine in the form of B-vitamins over the period of hospitalization. For example, B-vitamins are administered to mitigate against the risk of thiamine deficiency related neurodegenerative conditions (e.g. Wernicke-Korsakoff syndrome) that are known to occur in alcohol use disorder patients due to compromise of key enzymes involved in intermediary metabolism [12, 13]. Thiamine in the form of thiamine diphosphate (TDP) is required as a co-factor for pyruvate dehydrogenase (PDH) and alpha ketoglutaric acid dehydrogenase (KGDH), both key enzymes in Krebs cycle (Fig. 1). Thiamine also requires magnesium for absorption from the GI tract [14], activation to its active form (TDP) [15] and for optimal activity of thiamine dependent enzymes within the cell [16, 17]. Thiamine and/or magnesium deficiency may compromise these enzymes and result in altered metabolism of glucose and increased lactate production (Fig. 2) [18, 19]. Therefore, in the context of normal partial pressure tissue oxygen concentrations and in the absence of an inflammatory response or intense skeletal muscle activity (e.g. alcohol withdrawal seizure), thiamine and/or magnesium deficiency mediated increase in metabolism of glucose to lactate may represent pseudo-hypoxic disruption of normal glucose metabolism [7, 20].

Despite the prevalence of alcohol dependence in the western world, accurate data for thiamine deficiency is not available, with thiamine deficiency recorded as being between 30–80% in the AUD population [21, 22]. Furthermore, there is a paucity of accurate evidence to guide clinicians regarding the dose and duration of thiamine treatment or prophylaxis for Wernicke’s encephalopathy (WE) [22, 23]. It has been observed that WE can occur in the context of apparently normal blood thiamine levels [24]. Case reports have accrued over the past four decades, which point to a role for magnesium in patients who remain refractory to thiamine treatment, and respond to concurrent administration of intravenous magnesium [25–27]. Despite these case reports and the biochemical evidence in favor of an enhanced response to thiamine therapy when administered with magnesium [16, 17, 28, 29], the potential relationship between thiamine and magnesium has remained largely unexplored in a clinical context.

We recently reported in a retrospective study that patients who experienced an episode of alcohol withdrawal syndrome received thiamine supplementation although thiamine diphosphate (TDP) concentrations were not determined. In contrast, serum magnesium concentrations were measured and patients who had low serum magnesium concentrations (Mg^2+^ < 0.75 mmol/L) were rarely supplemented and were four times more likely to die at 1 year [9]. Furthermore, there was little supplementation of such low magnesium concentrations following discharge from hospital. Therefore, treatment of AUD, based on combined thiamine and magnesium supplementation is not well standardized in either the acute or chronic setting.

To validate the above findings, a prospective study was carried out of the relationship between circulating concentrations of thiamine and magnesium, the substrates and products of intermediary metabolism, the clinicopathological characteristics of AWS and 1-year mortality in patients with AWS.

## Patients and methods

The study took place in the Emergency Department of Glasgow Royal Infirmary (GRI) between December 2016 and February 2018. GRI is a university teaching hospital that serves an urban population with a high burden of socio-economic deprivation and offers the full spectrum of adult acute receiving specialties to patients over 16 years old [30, 31]. The study was approved by the West of Scotland Research Ethics Committee (WoS REC: 16/WS/0162).

**Fig. 3:**
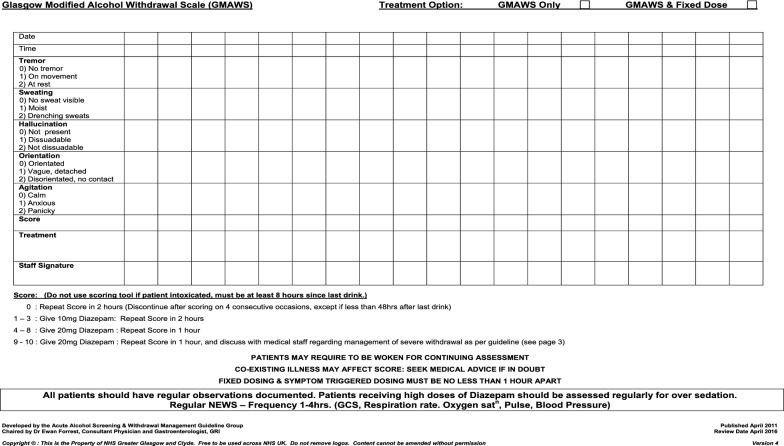
Glasgow Modified Alcohol Withdrawal Scale (GMAWS)

Age, sex, BMI and documented evidence of AWS were considered minimal criteria for inclusion (n = 127). Patients displaying clinical signs or reporting symptoms consistent with AWS at presentation to triage in the Emergency Department were approached in triage, or as soon as possible thereafter, regarding consent for participation in the study. As per routine clinical practice in the Emergency Department at GRI, patients were scored on the Glasgow Modified Alcohol Withdrawal Scale (GMAWS) and provided with standard benzodiazepine treatment for AWS (Fig. 3).

GMAWS is a validated score of severity of alcohol withdrawal syndrome that allocates a score (0–2) to five clinical characteristics (tremor, anxiety, sweating, orientation, hallucinations) (Fig. 3) [6]. GMAWS determines the dose of benzodiazepine treatment that is titrated and the time interval to re-administering the GMAWS scoring tool according to the score achieved (i.e. the severity of alcohol withdrawal). Diazepam (P.O.) or lorazepam (P.O. or I.V.) is administered as per GMAWS protocol. For the purposes of this study a ‘diazepam equivalent dose’ of benzodiazepine treatment was recorded, where diazepam 10 mg was equivalent to lorazepam 1 mg. A GMAWS ≥ 4 is considered severe, and is approximately equivalent to a Clinical Institute Withdrawal Assessment-Alcohol Revised (CIWA-Ar) scale ≥ 16 [6].

Bloods were drawn for routine biochemistry/haematology and study samples prior to administration of parenteral thiamine (Pabrinex^®^). Pabrinex^®^ is a two-vial multi-vitamin preparation that is administered intravenously in 100 ml normal saline over 30 min. Pabrinex^®^ contains thiamine hydrochloride 250 mg, nicotinamide 160 mg, pyridoxine hydrochloride 50 mg, riboflavin 4 mg and ascorbic acid 500 mg/10 mL [32]. A standard dose is 2 pairs of vials 1 and 2, administered TDS for 2 days [32–34].

Whole blood EDTA samples for thiamine diphosphate measurement were frozen at -70 °C and analyzed within 10 days of being drawn. Measurement of thiamine diphosphate in whole blood involved HPLC isocratic separation with post-column derivatization using sodium hydroxide and potassium ferricyanide and fluorescent detection. Results were expressed as nanogram of thiamine diphosphate per gram of haemoglobin (ng/gHb) [35].

A questionnaire was administered regarding recent weight loss, recent difficulty walking, smoking history, previous AWS episodes, previous AWS related seizures and use of ‘over the counter’ vitamin or magnesium supplements. Data relating to daily alcohol consumption were also recorded. Electronic patient records were also examined for data relating to past medical history and current medications.

Following completion of the study, electronic patient records were re-examined to record serum magnesium concentrations in patients during their next unplanned admission. Electronic patient records were also re-examined to record 1-year mortality in patients who had been entered into the study.

Standard thresholds were applied to the data. Continuous data were analyzed using the Mann–Whitney U test and categorical data were analyzed using the Chi squared test. Continuous data are presented as a median value and related inter-quartile range (IQR). Binary logistic regression analysis data was undertaken to examine the relationship between the severity of AWS (GMAWS </≥ 4) and independent variables. Data were analyzed in SPSS (Version 25.0. SPSS Inc., Chicago, IL, USA).

## Results

Only 3 patients who were screened for inclusion in the study at triage were found to have on-going active evidence of delirium. These patients were not recruited to the study, as they lacked capacity to consent. All patients (n = 127) recruited to the study fulfilled the criteria for inclusion with age, sex, BMI and documented evidence of alcohol withdrawal syndrome. Patients with 2 observations (n = 12) were excluded, leaving 115 patients for analysis.

The majority of patients (85%) reported previous episodes of alcohol withdrawal syndrome and had a documented history of previous alcohol withdrawal seizure (60%). Of the patients recruited to the study, 28% had suffered a tonic–clonic seizure within the 12-h period prior to presentation to the Emergency Department and 97% of these patients had a documented history of previous alcohol withdrawal seizures. Patients who had a previous history of AWS had higher GMAWS scores (p < 0.05), higher benzodiazepine requirement (p < 0.001) and more frequent previous admissions (p < 0.01) than those who had not previously experienced AWS.

Twenty-five patients (22%) recruited to the study progressed to develop delirium tremens and three patients (3%) suffered a tonic–clonic seizure during the course of the study related admission.

The clinicopathological characteristics are shown in Table 1. The majority of patients were < 50 years old (65%), male (76%), not underweight (80%), had a severe alcohol withdrawal score (GMAWS max score ≥ 4) (65%) and the total ‘diazepam-equivalent’ dose of benzodiazepine (BDZ) administered was more than 120 mg (51%). Of the laboratory analysis, the majority of patients had bilirubin (57%), alkaline phosphatase (67%), albumin (76%), CRP (56%), AST/ALT ratio (64%), glucose (66%), urea (67%), sodium (69%), potassium (81%), MCV (52%) and platelets (68%) within the laboratory reference interval. The majority of patients had been prescribed thiamine (69%) and a proton pump inhibitor (PPi) (55%).

**Table 1:** The relationship between clinicopathological characteristics and 1-year mortality in patients with AWS (n = 115)

	Alive (n = 102)	Dead (n = 13)	*p*-value^1^
Age (years)	48 (39–54)	48 (35–60)	0.915
Male/female	75/27	13/0	0.035
BMI (kg/m^2^)	22.6 (20.9–26.2)	21.9 (18.8–29.2)	0.473
Clinical			
Alcohol intake (U/week)^a^	205 (134–280)	280 (140–350)	0.390
GMAWS (at presentation)	4 (3, 4)	4 (4–5)	0.077
GMAWS max	4 (3–5)	5 (4–6)	0.090
GMAWS max (< 4/≥ 4)	38/63	1/12	0.033
BDZ total (mg)	130 (50–240)	230 (55–320)	0.148
Smoker (yes/no)	75/24	9/4	0.611
Recent weight loss (yes/no)	65/28	11/1	0.114
Gait disturbance (yes/no)	83/18	12/1	0.358
Laboratory			
Bilirubin (umol/L)	16 (10–28)	31 (13–75)	0.019
Alk phos (U/L)	106 (92–137)	130 (95–180)	0.154
Albumin (g/L)	39 (36–42)	37 (27.5–40)	0.106
CRP (mg/L)	4 (2–11)	17 (7–30)	0.004
AST	58 (34–98)	78 (29–131)	0.634
ALT	38 (25–61)	35 (18–53)	0.185
AST:ALT	1.6 (1.0–2.2)	1.9 (1.5–3.4)	0.038
Urea (mmol/L)	3.4 (2.6–4.8)	3.2 (2.5–5.4)	0.569
Sodium (mmol/L)	137 (134–140)	138 (136–141)	0.280
Potassium (mmol/L)	4.0 (3.7–4.4)	4.0 (3.5–4.5)	0.555
Serum magnesium (mmol/L)	0.72 (0.62–0.80)	0.53 (0.48–0.72)	0.006
Plasma H^+^ (nmol/L)	37 (33–40)	33 (27–40)	0.175
Plasma HCO_3_^−^ (mmol/L)	27 (25–32)	28 (24–35)	0.745
MCV (fl)	95.4 (90.6–99.7)	100.4 (93.1–106.7)	0.059
Platelets (10^9^/L)	239 (143–295)	175 (78–240)	0.092
Whole blood TDP (ng/gHb)	595 (509–825)	670 (391–791)	0.720
LDH (U/L)	257 (208–325)	272 (220–369)	0.373
Glucose (mmol/L)	5.8 (5.3–7.5)	6.9 (5.9–7.7)	0.183
Lactate (mmol/L)	2.3 (1.5–4.2)	3.8 (2.6–7.2)	0.011
Follow-up ‘next admission’ serum magnesium (mmol/L)	0.68 (0.59–0.76)	0.61 (0.49–0.67)	0.020
Medications			
Thiamine (yes/no)	77/23	2/11	0.978
Magnesium (yes/no)	8/88	13/0	0.282
PPi (yes/no)	59/40	4/9	0.506
Diuretic (yes/no)	5/91	4/9	0.002
Admission profile			
Admitted within previous month (yes/no)	23/75	3/10	0.975
ED presentations within 12 months	2 (1–5)	3 (2–7)	0.315
Total number of admissions	14 (6–32)	14 (4–57)	0.466
Length of stay (days)	4 (3–7)	8 (5–10)	0.027

^1^Continuous data were analysed with Mann–Whitney U test and categorical data were analysed with Chi squared test

^a^Alcohol intake–alive: n = 54; dead: n = 3

The majority of patients had whole blood thiamine diphosphate concentrations (59%) within the normal reference interval (275–675 ng/gHb) and 40% of patients had whole blood thiamine diphosphate concentrations above the reference interval (> 675 ng/gHb). One-third of patients (33%) (n = 14) who had whole blood thiamine diphosphate concentrations above the reference interval (> 675 ng/gHb) (n = 42) at the time of study recruitment had been admitted to hospital and treated with parenteral thiamine within 1 month prior to the study related admission. The majority of patients (73%) had been prescribed long-term oral thiamine supplementation (50 mg or 100 mg TDS) in the community prior to entering the study. Only one of the patients (< 1%) entered into the study had a whole blood thiamine concentration below the reference interval (< 275 ng/gHb).

**Fig. 4:**
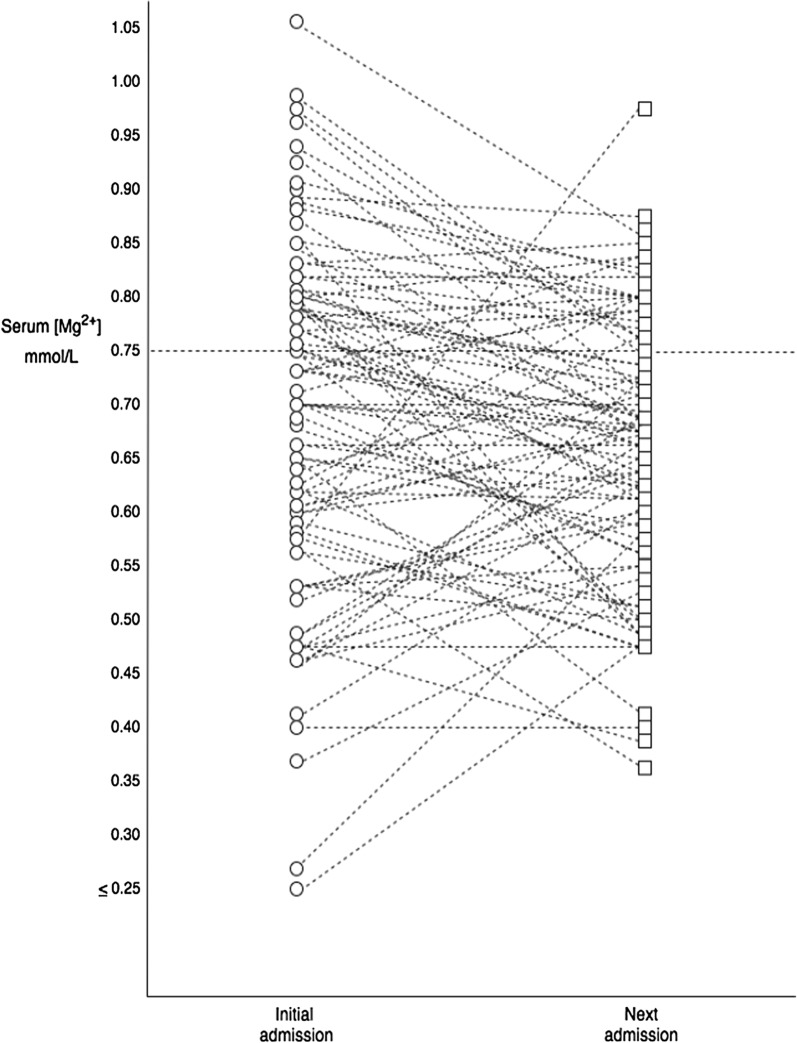
Diagrammatic representation of relative change between initial serum magnesium concentrations and ‘next admission’ serum magnesium concentrations in prospective observational study of patients presenting to the Emergency Department with alcohol withdrawal syndrome (n = 81)

In contrast, the majority of patients had serum magnesium concentrations that could be considered to be low (< 0.75 mmol/L; 60%) and had not been prescribed magnesium (93%). When patients from this cohort (n = 81) were followed up at their next admission, the majority of patients (74%) still had serum magnesium concentrations below the reference interval (< 0.75 mmol/L) (median = 0.67 mmol/L, IQR 0.59–0.76 mmol/L, median time interval between samples = 126 days) (see Tables 1, 2; Fig. 4).

**Table 2:** The relationship between circulating magnesium concentrations and clinicopathological characteristics in patients with alcohol withdrawal syndrome (n = 111)

	Serum magnesium (mmol/L)		*p*-value^1^
	≥ 0.75 (n = 46)	< 0.75 (n = 65)	
Age (years)	46 (35–49)	48 (40–54)	0.139
Male/female	33/13	53/12	0.226
BMI (kg/m^2^)	22.5 (20.9–24.8)	22.7 (20.5–27.1)	0.571
Dead at 1-year (yes/no)	2/44	11/54	0.043
Clinical			
Alcohol intake (U/week)^a^	210 (145–280)	168 (42–420)	0.259
GMAWS (at presentation)	3 (2–4)	4 (3–5)	0.006
GMAWS max	3 (2–4)	4 (4–6)	0.001
BDZ total (mg)	100 (40–210)	180 (50- 290)	0.335
Smoker (yes/no)	31/13	50/14	0.368
Recent weight loss (yes/no)	29/14	46/14	0.302
Gait disturbance (yes/no)	35/11	56/8	0.120
Laboratory			
Bilirubin (umol/L)	12 (8–20)	17 (13–41)	< 0.001
Alk phos (U/L)	101 (90–138)	114 (94–140)	0.430
Albumin (g/L)	39 (36–43)	38 (36–41)	0.168
CRP (mg/L)	4.5 (1–10.5)	5 (2–14.3)	0.303
AST	44 (31 75)	66 (41–213)	0.015
ALT	37 (24–61)	38 (25–60)	0.854
AST:ALT	1.4 (1.0–1.8)	1.6 (1.3–2.8)	0.004
Urea (mmol/L)	3.3 (2.6–5.2)	3.3 (2.5–4.9)	0.593
Sodium (mmol/L)	138 (134–141)	138 (134–139)	0.422
Potassium (mmol/L)	4.1 (3.7–4.5)	4.0 (3.6–4.3)	0.437
Plasma H^+^ (nmol/L)	39 (35–40)	36 (31–39)	0.013
Plasma HCO_3_^−^ (mmol/L)	29 (24–33)	27 (25–31)	0.812
MCV (fl)	95.2 (90.9–99.6)	95.7 (91.2–100.4)	0.418
Platelets (10^9^/L)	256 (165–312)	204 (117–282)	0.020
Follow-up ‘next admission’ serum magnesium (mmol/L)	0.73 (0.66–0.79)	0.61 (0.55–0.68)	< 0.001
Whole blood TDP (ng/gHb)	642 (500–855)	591 (501–775)	0.305
LDH (U/L)	240 (209–308)	269 (213–358)	0.333
Glucose (mmol/L)	5.8 (5.4–7.2)	6.2 (5.3–7.9)	0.477
Lactate (mmol/L)	2.3 (1.3–3.4)	2.9 (1.9–5.9)	0.023
Medications			
Thiamine (yes/no)	34/12	51/13	0.478
Magnesium (yes/no)	5/41	3/57	0.259
PPi (yes/no)	26/20	39/24	0.573
Diuretic (yes/no)	1/44	8/53	0.048
Admission profile			
Admitted within previous month (yes/no)	14/31	11/52	0.077
ED presentations within 12 months	3 (1–7)	2 (0–36)	0.923
Total number of admissions	15 (6–33)	14 (0–172)	0.646
Length of stay (days)	4 (3–7)	5 (1–29)	0.171

^1^Continuous data were analysed with Mann–Whitney U test and categorical data were analysed with Chi squared test

^a^Alcohol intake: Mg^2+^ ≥ and < 0.75 mmol/L: n = 22; n = 32

The majority of patients (66%) were found to have plasma lactate concentrations above 2.0 mmol/L. When patients who had experienced a seizure in the 12-hour period prior to study enrolment (n = 29) were excluded from the analysis, the majority (60%) of the remaining patients (n = 74) had plasma lactate concentrations above 2.0 mmol/L. The median plasma lactate concentration for this sub-set of patients (i.e. non-seizure related alcohol withdrawal syndrome presentations) was 2.4 mmol/L (IQR 1.6–4.5 mmol/L). The median plasma lactate concentration for patients recruited to the study with alcohol withdrawal syndrome who had experienced a seizure within 12 h of presentation (n = 29) was 2.5 mmol/L (IQR 1.7–14.6 mmol/L). Serum magnesium concentrations were significantly associated with plasma lactate concentrations (r_s_ = − 0.234, p < 0.05).

At 1 year, 13 patients had died giving a mortality rate of 11%. The relationship between 1-year mortality and clinicopathological characteristics are shown in Table 1. On univariate analysis, male gender (p < 0.05), BMI < 20 kg/m^2^ (p < 0.01), GMAWS max ≥ 4 (p < 0.05), elevated plasma lactate (p < 0.01), low serum albumin (p < 0.05), elevated serum CRP (p < 0.05), low serum magnesium at time of recruitment to study (p < 0.05) and low serum magnesium concentration at next admission (p < 0.05) were associated with death at 1-year.

The relationship between low serum magnesium concentrations and clinicopathological characteristics is shown in Table 2. Low serum magnesium concentrations were associated with a higher GMAWS score at presentation (p < 0.01), higher GMAWS max (p = 0.001), elevated hydrogen ion concentrations (> 45 nmol/L) (p < 0.05) and death at 1-year (p < 0.05).

Binary logistic regression analysis confirmed that patients who had low serum magnesium concentrations (< 0.75 mmol/L) were 3 times more likely to have a severe episode of AWS (GMAWS ≥ 4) when compared to those patients who had serum magnesium concentrations within the normal reference interval (O.R. = 3.0, 95% CI (1.1–8.0), p < 0.05).

## Discussion

The results of the present prospective study show that the majority of patients with AWS had thiamine concentrations within or above the normal interval. In contrast, the majority of patients had low serum magnesium concentrations at presentation. Furthermore, low serum magnesium concentrations (< 0.75 mmol/L) were associated with increased risk of mortality at 1-year among patients admitted to hospital with AWS. These results highlight the importance of a low serum magnesium concentration, and its lack of treatment, in outcome of patients presenting with AWS.

It was of interest that only 1 patient (< 1%) in the cohort (n = 115) had a baseline circulating thiamine diphosphate measurement below the reference interval (< 275 ng/gHb). Indeed, almost 40% of patients had circulating thiamine diphosphate concentrations above the upper limit of the reference interval (> 675 ng/gHb), and had been prescribed thiamine (73%). In contrast, approximately 60% of patients had low serum magnesium concentrations (< 0.75 mmol/L) and had not been prescribed magnesium (93%). Furthermore, serum magnesium concentrations remained low (< 0.75 mmol/L) when measured during the next acute admission (Fig. 4). These results are consistent with a recent retrospective study (n = 380) that reported a prevalence of low serum magnesium concentrations (< 0.75 mmol/L) (> 60%) and 17% 1-year mortality for patients presenting with AWS [9].

The majority of patients (60%) who had circulating thiamine diphosphate concentrations above the reference interval (> 675 ng/gHb) at the time of study recruitment had been admitted to hospital and treated with parenteral thiamine within 1 month prior to the study related admission. However, in routine clinical practice thiamine concentrations are rarely measured, and if so, results are only available weeks later. Hence, the established clinical appreciation of the importance of thiamine deficiency in exacerbating symptoms in patients with AWS would appear to have resulted in the empirical administration of commercially available thiamine (Pabrinex^®^) to patients with AWS admitted to the general hospital setting [12]. In the present cohort of patients, community treatment with oral thiamine combined with the intravenous administration of thiamine during frequent hospital admissions may account for the normal and high circulating concentrations of whole blood thiamine diphosphate observed. In contrast, in the case of magnesium, although results are obtained within minutes/hours of sampling, low serum magnesium concentrations were not routinely supplemented. The infrequency of magnesium supplementation in the context of low serum magnesium concentrations may reflect a lack of clinician awareness that exists regarding the clinical significance and prevalence of magnesium deficiency among AUD patients. This in turn likely reflects a paucity of evidence regarding the prevalence of magnesium deficiency and other micronutrient deficiencies (e.g. vitamin C) among the AUD patient population [36]. Although it has been understood biochemically for decades that thiamine is dependent upon magnesium for optimal metabolism, in the absence of clinical data this has not translated into widespread clinical practice to date.

The measurement and interpretation of thiamine levels is quite problematic and the ideal method remains contentious [37]. Indeed, Cook et al. observed in their review that it was the activity of thiamine, which was relevant, rather than the finite mass available [38]. Erythrocyte transketolase activity (ETKA) is a functional marker of thiamine dependent enzyme activity which was commonly used to measure thiamine status until the late 1990s, however this method of routine measurement of thiamine status fell out of favor due to difficulty with standardization, inter-laboratory variation and the advent of high performance liquid chromatography (HPLC), which enabled direct measurement of TDP mass [39, 40]. Direct measurement of erythrocyte and/or whole blood thiamine concentrations is now more widely available [35] and has the advantage of remaining consistent in the context of the systemic inflammatory response [41, 42]. Nonetheless, it is of note that significant enhancement of basal erythrocyte transketolase activity (ETKA) has been demonstrated in response to co-administration of thiamine and magnesium, as compared to thiamine alone, in a cohort of alcohol dependent patients [16].

On the basis of the present results, it may be proposed that further studies to elucidate the pharmacokinetics of parenteral thiamine supplementation are required to clarify the safe therapeutic window during which patients with AWS may not require further treatment with parenteral thiamine. The basis of Wernicke’s encephalopathy (WE) is not clear [18]. However, it is clear that WE is exacerbated by thiamine deficiency [33, 43]. Under normal physiological and nutritional conditions, the average adult human has approximately a 3-week reserve of thiamine in the liver and skeletal muscle. These reserves become rapidly depleted in alcohol related disease [43, 44]. Therefore, given the present results, it is likely that these patients were compliant with thiamine supplementation as routinely prescribed and confirm the success of a therapeutic strategy for a patient group who are known to be at high risk of thiamine deficiency. Overall, the clinical consequences of under-treating thiamine deficiency are potentially devastating, while treatment with parenteral thiamine appears to have a good safety profile and is relatively inexpensive. However, it is not clear whether chronically high circulating thiamine may have a detrimental effect on health.

The basis of the finding that low serum magnesium concentrations (< 0.75 mmol/L) were independently associated with more severe alcohol withdrawal syndrome (GMAWS ≥ 4) is not clear. However, the present results are in keeping with in vitro data that confirms the role of magnesium in stabilisation of the NMDA receptor [45]. NMDA receptors require binding of glutamate and glycine, combined with the relief of the voltage-dependent magnesium block, to open the calcium ion conductive pore [45]. The association between higher maximal AWS scores (GMAWS max) and low serum magnesium concentrations may reflect the reliance of the NMDA receptor on the stabilizing effect of magnesium (see Table 2) [46, 47]. Hence, a biochemical cause for AWS related seizure activity may be converted to a structural focus for seizure activity and result in lower subsequent seizure thresholds [46]. This process of alcohol withdrawal ‘seizure kindling’ may be reflected in the strong association between a documented history of previous alcohol withdrawal seizures that was noted in a high proportion of patients (97%) who had sustained an alcohol withdrawal seizure in the 12-h period prior to recruitment to the study.

The present study also reports data relating to the interrelationship between thiamine and magnesium status in intermediary metabolism, as reflected in plasma lactate concentrations. Serum magnesium concentrations were significantly associated with plasma lactate concentrations. One possible explanation of the association between low serum magnesium concentrations and raised plasma lactate concentrations, may be that magnesium deficiency mediated compromise of PDH activity can result in pyruvate being unable to gain access into the mitochondrion for conversion to acetyl-CoA and thereby onto Krebs cycle (Figs. 1, 2) [28]. The resulting ‘glut’ of pyruvate in the cytosol may trigger up-regulation of LDH activity [48]. LDH mediates the increased production of lactate, which accumulates in the cytosol [49]. This may be considered the suboptimal metabolism of glucose i.e. a pseudo-hypoxic ‘dirty burn’ (Fig. 2) [7]. Given the requirement of PDH for TDP and magnesium, chronic ingestion of excessive amounts of alcohol in the context of a micronutrient poor diet may therefore result in pseudo-hypoxic dysregulation of glucose metabolism (i.e. ‘dirty burn’ metabolism) with increased lactate production (Fig. 2) [7, 50].

Interestingly, elevated plasma lactate was significantly associated with 1-year mortality and this may reflect a chronic loss of oxidative resilience among this patient group. Alcohol related cirrhotic liver disease is reported to cause increased splanchnic and systemic plasma lactate concentrations [51], however associations between plasma lactate and serum bilirubin concentrations or plasma lactate concentrations and AST:ALT ratio > 2 were not significant. Overall, this series of patients would appear to represent the largest cohort of AWS patients to have non-seizure related elevated plasma lactate concentrations recorded to date.

## Limitations

The present study sample may not be representative of all patients experiencing alcohol use disorder. Patients who experience AWS represent a group of AUD patients who have a very high level of dependence and of withdrawal complications. Indeed, the patients described in the present study represent a group of AUD patients who have been treated with previous thiamine supplementation. Furthermore, it is worth considering that in the context of such high dose supplementation, other B-vitamins apart from thiamine (e.g. nicotinamide, pyridoxine and riboflavin) may also be within their normal interval ranges for those patients who receive frequent high dose parenteral therapy. Another important limitation of this study is the lack of measurement of other micronutrients e.g. B-vitamins, vitamin C and trace elements e.g. selenium and zinc. Further studies that present data relating to more comprehensive nutritional assessment are required in order to better nutritionally phenotype these individuals.

Also, the relatively small sample size (n = 115) may be considered a limitation of this study. Nonetheless, this sample presenting prospective data relating to the clinicopathological characteristics of patients experiencing AWS is one of the largest reported to date.

The underlying mechanisms that could explain a relation between low circulating magnesium concentrations and increased all-cause mortality remain to be established. For example, it may be postulated that those patients with low circulating magnesium concentrations may have had poorer access to care, therefore they were less likely to be offered supplementation and may have died sooner, but without any direct link between these two conditions. To establish the potential benefit of magnesium supplementation, it will be necessary to carry out a randomised controlled trial of magnesium supplementation in patients with AWS.

## Conclusion

The prevalence of low circulating thiamine concentrations were rare and it was regularly prescribed in patients with AWS. In contrast, low serum magnesium concentrations were common and not prescribed. Low serum magnesium was associated more severe AWS and increased 1-year mortality.

## Data Availability

Data will be made available upon request to the corresponding author.

## Abbreviations

AWS: alcohol withdrawal syndrome
TDP: whole blood thiamine diphosphate
ng/gHb: nanogram per gram of haemoglobin
Mg^2+^: serum magnesium
mmol/L: millimole per litre
GMAWS: glasgow modified alcohol withdrawal scale ()
BMI: body mass index
CRP: C-reactive protein
NMDA: N-methyl-d-aspartate
GABA: gamma-aminobutyric acid
CIWA-Ar: Clinical Institute Withdrawal Assessment-Alcohol Revised
PPi: proton pump inhibitor
AST: aspartate transaminase
ALT: alanine aminotransaminase
LDH: lactate dehydrogenase
MCV: mean corpuscular volume
r_s_: Spearman correlation
